# A Wheat R2R3-type MYB Transcription Factor TaODORANT1 Positively Regulates Drought and Salt Stress Responses in Transgenic Tobacco Plants

**DOI:** 10.3389/fpls.2017.01374

**Published:** 2017-08-08

**Authors:** Qiuhui Wei, Qingchen Luo, Ruibin Wang, Fan Zhang, Yuan He, Yang Zhang, Ding Qiu, Kexiu Li, Junli Chang, Guangxiao Yang, Guangyuan He

**Affiliations:** The Genetic Engineering International Cooperation Base of Chinese Ministry of Science and Technology, Key Laboratory of Molecular Biophysics of Chinese Ministry of Education, College of Life Science and Technology, Huazhong University of Science and Technology Wuhan, China

**Keywords:** wheat, abiotic stress, MYB, antioxidation system, stress related genes

## Abstract

MYB transcription factors play important roles in plant responses to biotic and abiotic stress. In this study, *TaODORANT1*, a R2R3-MYB gene, was cloned from wheat (*Triticum aestivum* L.). TaODORANT1 was localized in the nucleus and functioned as a transcriptional activator. *TaODORANT1* was up-regulated in wheat under PEG6000, NaCl, ABA, and H_2_O_2_ treatments. *TaODORANT1*-overexpressing transgenic tobacco plants exhibited higher relative water content and lower water loss rate under drought stress, as well as lower Na^+^ accumulation in leaves under salt stress. The transgenic plants showed higher CAT activity but lower ion leakage, H_2_O_2_ and malondialdehyde contents under drought and salt stresses. Besides, the transgenic plants also exhibited higher SOD activity under drought stress. Our results also revealed that *TaODORANT1* overexpression up-regulated the expression of several ROS- and stress-related genes in response to both drought and salt stresses, thus enhancing transgenic tobacco plants tolerance. Our studies demonstrate that TaODORANT1 positively regulates plant tolerance to drought and salt stresses.

## Introduction

Plants are very often subjected to unfavorable environmental conditions, such as high salinity, drought, and extreme temperatures, which adversely affect plant growth, development, and productivity. To adapt to such environmental conditions, complex response mechanisms have been evolved in plants, including transcriptional regulation networks for the transduction of stress signals. The implementation of these intricate networks depends on the participation of various transcription factors (TFs), such as MYB, NAC, AP2/ERF, bZIP, bHLH, and WRKY (Qin et al., 2011; Chen et al., 2012).

The MYB superfamily plays a crucial role in abiotic stress responses. The first known MYB gene, isolated from maize (*Zea mays*) and designated *COLORED1(C1)*, is required for anthocyanin synthesis in the aleurone of maize kernels (Paz-Ares et al., 1987). The MYB superfamily has been identified and analyzed in Arabidopsis and rice (Dubos et al., 2010; Katiyar et al., 2012). The MYB superfamily is divided into four subgroups based on the number of conserved MYB-domain repeats, i.e., a single or partial MYB repeat protein MYB1R, two repeats protein R2R3-MYB, three repeats protein MYB3R, and four R1/R2-like repeats protein 4R-MYB (Dubos et al., 2010). Several lines of evidence demonstrate that numerous R2R3-MYB proteins function in response to abiotic stress. In Arabidopsis, *AtMYB96* overexpression enhances drought tolerance by integrating the ABA and auxin signaling pathways, while at the same time improving freezing and drought tolerance by regulating the target gene *LTP3* (Seo et al., 2009; Guo et al., 2013). Meanwhile, AtMYB44 and AtMYB60 participate in plant responses to drought stress by regulating stomatal movement (Cominelli et al., 2005; Jung et al., 2008). *AtMYB20*-overexpressing Arabidopsis plants display salt-stress tolerance but susceptible to desiccation (Cui et al., 2013; Gao et al., 2014). AtMYB14 and AtMYB15 are known to participate in plant adaptation to freezing temperatures (Agarwal et al., 2006; Chen et al., 2013). In rice (*Oryza sativa*), the R2R3-type MYB gene *OsMYB91* is involved in salt-stress tolerance and plant growth (Zhu et al., 2015). OsMYB2 has been implicated in tolerance to salt, freezing, and dehydration (Yang et al., 2012). In addition, MYB3R and MYB-related proteins, such as OsMYB511, OsMYB48-1, OsMYB4, and OsMYB3R-2, are known to be involved in stress adaptation (Dai et al., 2007; Pasquali et al., 2008; Xiong et al., 2014; Huang P. et al., 2015). GbMYB5 improves drought tolerance in transgenic cotton and tobacco plants (Chen et al., 2015). Overexpression of *LeAN2*, initially isolated from tomato plants, positively regulates responses to chilling and oxidative stresses in tobacco plants (Meng et al., 2014).

Given that wheat (*Triticum aestivum* L.) is a major grain crop, studies on the response of wheat to various adverse environments have attracted increasing attention. Significant progress in wheat genome sequencing has been achieved in recent years, laying the foundations for successful gene identification and isolation (Jia et al., 2013; Ling et al., 2013; Choulet et al., 2014; Mayer et al., 2014). Although many *MYB* genes have been identified in wheat, only a few have so far been characterized. Abiotic stress can induce *TaMyb1* expression in the roots of wheat plants (Lee et al., 2006). Of the seven *MYB* gene fragments that were identified by Rahaie et al. (2010), *TaMYBsdul* was suggested to improve salt and drought tolerance in wheat (Rahaie et al., 2010). *TaPIMP1* overexpression enhances disease, drought, and salt stress resistance in both transgenic tobacco and wheat (Liu et al., 2011; Zhang Z.Y. et al., 2012). Arabidopsis plants that overexpress *TaMYB19* also show an improved tolerance to abiotic stress (Zhang et al., 2014).

In the present study, *TaODORANT1*, a R2R3-type MYB transcription factor gene, was cloned from wheat (*T. aestivum* cv. Chinese Spring). Gene expression profiles revealed that *TaODORANT1* was up-regulated under high salinity, PEG6000, H_2_O_2_, and ABA treatments. *TaODORANT1* overexpression conferred drought and salt tolerance to transgenic tobacco plants.

## Materials and Methods

### Plant Material and Treatments

Wheat (*T. aestivum* cv. Chinese Spring) was used in this study. Seeds were germinated in the dark and were cultivated in a greenhouse (12 h light/12 h dark cycle at 22°C). For the organ expression assay, roots, stems, and leaves were collected from 14-day-old seedlings. Mature roots, mature stems, mature leaves, stamens, and pistils were obtained from wheat plants at flowering stage. For drought and salt stress treatments, 14-day-old seedlings were cultured in solutions that contained 20% PEG6000 (w/v) or 200 mM NaCl. For ABA and hydrogen peroxide (H_2_O_2_) treatments, seedling roots were dipped into 100 μM ABA or 10 mM H_2_O_2_ solution, and the seedling leaves were sprayed with the same solutions. All samples were collected at the time points indicated, frozen in liquid nitrogen, and stored at -80°C for subsequent RNA extraction.

### Cloning and Bioinformatic Analysis of *TaODORANT1*

All known Arabidopsis and rice MYB protein sequences were acquired from relevant databases^1^^,^^2^. Expression profiles were predicted with PLEXdb^3^. The accuracy and integrity of cDNA were verified with Ensembl Plants^4^ and IWGSC^5^. cDNA sequences were amplified with primer pairs (Supplementary Table S1) that were designed using the software Primer Premier 5 (PREMIER Biosoft, Palo Alto, CA, United States). Templates were synthesized from RNA mixtures that were extracted from wheat organs at different developmental stages and from seedlings that had been treated with NaCl, PEG6000, ABA and H_2_O_2_. Polymerase chain reaction (PCR) products were sequenced (AuGCT Biotech, Beijing, China) with *TaODORANT1* (accession no. KY013614) as the target gene. The *TaODORANT1* promoter fragment was cloned *via* PCR with the primers listed in Supplementary Table S1. Promoter sequence was analyzed using the software PlantCARE^6^. Homologous TaODORANT1 protein sequences were collected from NCBI database^7^ and were aligned using the software ClustalX (Conway Institute, Dublin, Ireland). A phylogenetic tree was generated by using MEGA 5 software coupled with Neighbor-Joining method (Tamura et al., 2011).

### Subcellular Localization

In order to detect the subcellular localization of TaODORANT1 *in vivo*, pMD18-T vector was constructed with the maize *ubiquitin* promoter and green fluorescent protein (*GFP*) gene to form the expression vector pMD18-ubi-GFP. Then, the open reading frame (ORF) of *TaODORANT1* was amplified using specific primers that contained *Hin*dIII*/Spe*I restriction sites (Supplementary Table S1). The amplified *TaODORANT1* ORF was fused to the 5′-terminal end of the *GFP* gene in the pMD18-ubi-GFP vector to generate a recombinant vector. The recombinant vector ubiqutin::TaODORANT1-GFP and the control vector pMD18-Ubi-GFP were transformed into onion epidermal cells, respectively, *via* particle bombardment. The results were observed with fluorescence microscopy (IX71, Olympus, Japan).

### Transactivational and Binding Activity Analysis

Transcriptional activity was investigated using the Clontech Matchmaker^TM^ Yeast One-Hybrid system (TBUSA, Mountain View, CA, United States), a GAL4-based yeast one-hybrid system. The ORF at 1–795 base pair (bp) as well as various truncated ORFs at 1–360, 186–795, 361–795, and 513–795 bp of *TaODORANT1* were amplified by PCR using specific primers that contained *Eco*RI/*Bam*HI restriction sites (Supplementary Table S1). These fragments were then inserted into the pGBKT7 vector. An empty pGBKT7 vector was used as the negative control plasmid. For the binding activity assay, *TaODORANT1* and three typical MYB binding motifs were introduced into the pGADT7 and pHIS2 plasmids (Supplementary Table S1). The recombinants were co-transformed into the yeast strain Y187. Yeast transformation and screening were performed in accordance with the users’ manual (Clontech, United States).

### Expression Analysis of *TaODORANT1* in Wheat

Total RNA was extracted from different samples with a Plant Total RNA Extraction Kit (Zoman, Beijing, China). First-strand cDNA was synthesized with the FastQuant RT Kit (TIANGEN, Beijing, China). Quantitative real-time PCR (qRT-PCR) was performed with SuperReal PreMix Plus Kits (TIANGEN, Beijing, China). Each reaction solution contained 5 μl of 2 × SuperReal PreMix, 1 μl of gene-specific primers, and 1 μl of cDNA (about 50 ng), and was added with ddH_2_O to a final volume of 10 μl. The PCR program was as follows: 95°C for 15 min; 50 cycles of 95°C for 10 s; 55°C for 20 s; 72°C for 30 s. qRT-PCR was performed with the CFX Connect Real-Time System (Bio-Rad, Hercules, CA, United States). Expression data were analyzed with the comparative 2^-ΔΔ*C*_T_^ method (Livak and Schmittgen, 2001). The primers used in this assay are listed in Supplementary Table S1. The housekeeping wheat gene *actin* (accession no. AB181991.1) was used as the internal control.

### Plant Transformation

To generate transgenic tobacco plants that overexpressed *TaODORANT1*, the ORF that contained the terminator codon was cloned into the pBI121 vector under the control of the cauliflower mosaic virus *35S* promoter with *Xba*I/*Bam*HI restriction sites. The pBI121-TaODORANT1-GFP constructs and pBI121-GFP vector were transformed into *Agrobacterium tumefaciens* strain EHA105. Transformation was accomplished using the *A. tumefaciens-*mediated leaf disk method (Horsch et al., 1985). Eight independent transgenic T_2_ lines were obtained. The expression level of *TaODORANT1* in each line was examined by RT-PCR.

### Southern Blotting Analysis

Genomic DNAs of wildtype (WT) and transgenic tobacco plants were extracted by CTAB method (Staccy and Isaac, 1994), and were digested by restriction enzyme *Hin*dIII. Then, the digested gDNAs were separated by electrophoresis and transferred to Hybond-N+ membrane according to the manufacturer’s protocol (Roche). Membrane was hybridized with digoxigenin (DIG) labeled probe (*TaODORANT1*). Finally, hybridized probe DNA was observed by exposure to Kodak double-emulsion films. The probe primers were listed in Supplementary Table S1.

### Stress Tolerance Analysis of the Transgenic Plants

Wildtype, vacant vector control (VC), and overexpression (OE) lines were used to analyze stress tolerance. Seeds were surface-sterilized with 75% ethanol for 1 min and 10% H_2_O_2_ for 8 min. The seeds were then sown on 1/2 Murashige and Skoog (MS) medium and incubated in a growth chamber (16 h light/8 h dark cycle at 22°C) for 10 days. The seedlings were transferred to 1/2 MS media that contained 150/300 mM mannitol or 150/200 mM NaCl for osmotic and salt stress assays. Root length was measured after 10 days of treatment. To analyze the stress tolerance of transgenic plants, 2-week-old seedlings were planted in pots that were filled with an equal quantity of moisture and soil and grown in a greenhouse under a 12 h light/12 h dark cycle at 22°C. In each biological replicate, about 50 tobacco plants (10 pots) of each line were treated. For the drought stress tolerance assay of transgenic plants, 4-week-old plants were withheld water for 27 days and then re-watered for 1 week. For the salt stress tolerance assay of transgenic plants, 3-week-old plants grown in pots were treated in a container containing 2 L 500 mM NaCl for 19 days. Supplemental NaCl solution was added to the container every 3 days throughout the treatment period. The water loss assay was performed as described by Huang Q. et al. (2015). The stomatal aperture assay was accomplished in accordance with Yan et al. (2014) with slight modifications: the duration of dehydration and ABA (50 μM) treatment were modified to 40 min and 1 h, respectively.

### Measurement of RWC, IL, MDA, and H_2_O_2_ Contents and CAT, SOD, and POD Enzyme Activities

Leaves (second or third leaves from the top) with similar size were sampled from the WT, VC, and OE plants that were exposed to drought and salt stress. Relative water content (RWC) and ion leakage (IL) were determined as described by Hu et al. (2013). Malondialdehyde (MDA) accumulation was measured using the thiobarbituric acid-based method with an MDA assay kit (Jiancheng, Nanjing, China). H_2_O_2_ content as well as CAT, SOD, and POD activities were measured by spectrophotometry using the corresponding assay kits (Jiancheng, Nanjing, China). Superoxide anion radicals (O_2_^-^) and H_2_O_2_ were histochemically detected with the DAB and NBT staining method in accordance with the procedure by Hu et al. (2013).

### Measurement of Chlorophyll Content and Na^+^ and K^+^ Concentrations

Chlorophyll content was measured as described by Arnon (1949). Na^+^ and K^+^ concentrations were detected with plant total Na^+^ and K^+^ kits (Keming, Suzhou, China) in accordance with the kits’ protocols. Leaves were washed with Milli-Q water, dried at 70°C, and then ground to power. The powdered samples were used for ion concentration measurement. To measure Na^+^ concentration, Na^+^ was reacted with potassium pyroantimonate to form sodium pyroantimonate sediment under special conditions. To measure K^+^ concentration, K^+^ was reacted with sodium tetraphenylborate to form potassium tetraphenylborate sediment under special conditions. Then, the turbidity of the test solution was measured at 520 nm using a spectrophotometer.

### Expression Assay of Stress-Related Genes

Two-week-old tobacco seedlings were treated on 1/2 MS media with 300 mM mannitol or 150 mM NaCl for 1 week. Then the total RNA of the seedlings was extracted to analyze the expression of stress-induced genes by qRT-PCR. The specific primers used are listed in Supplementary Table S1. The *Ntubiqutin* gene was selected as the internal control.

### Statistical Analysis

Statistical analysis was performed with SPSS (IBM Analytics, New York, NY, United States) and Student’s *t*-test.

## Results

### Identification of *TaODORANT1*

**FIGURE 1:**
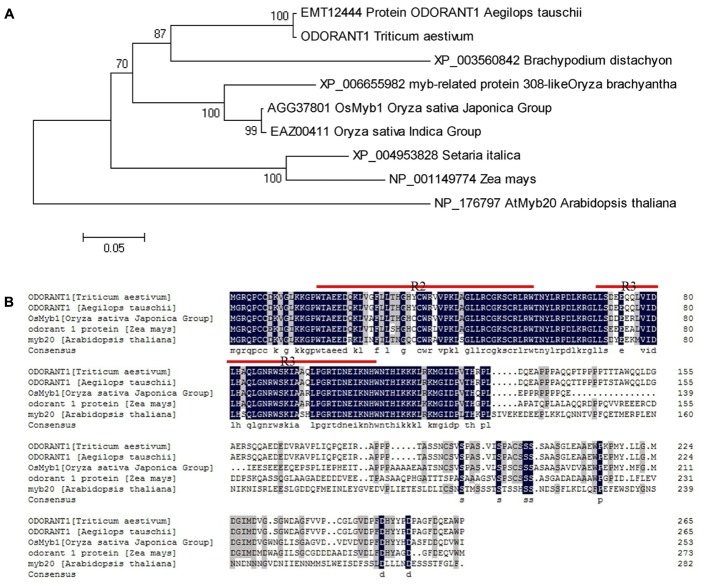
Sequence and phylogenetic tree analysis of TaODORANT1. **(A)** Phylogenetic relationship of TaODORANT1 with its orthologs from other plant species. **(B)** Sequence alignment of TaODORANT1 and homologous proteins from other plant species. The black background represents identical amino acid residues in the aligned sequences. Red straight lines indicate the conserved domains of R2R3-MYB.

Wheat MYB expressed sequence tags (ESTs) were obtained by searching the NCBI UniGene database^8^ with known Arabidopsis and rice MYB sequences listed in Supplementary Table S3. The obtained ESTs were assembled into longer cDNA sequences. A cDNA sequence was selected based on the data acquired from an expression database^3^ (Supplementary Figure S3). The accuracy and integrity of this cDNA sequence were confirmed by Ensembl Plants^4^ and IWGSC^5^. The putative *MYB* gene was identified and cloned from wheat. The cloned cDNA sequence is 1,039-bp long containing a 798-bp ORF, which was predicted to encode a 265 amino acid protein with a relative molecular mass of 29.211 kDa and an isoelectric point of 6.17. A phylogenetic tree was generated with this MYB sequence and its orthologs from different plant species. Phylogenetic analysis showed that the MYB sequence had the closest relationship with protein ODORANT1 from *Aegilops tauschii* (**Figure 1A**). Therefore, the putative MYB gene was designated *TaODORANT1*. Aligning TaODORANT1 with homologous proteins from other plant species revealed two conversed repeats in the DNA-binding domain, which classified TaODORANT1 to the R2R3-type MYB subfamily (**Figure 1B**).

### Subcellular Localization and Transactivation Activity Analysis of TaODORANT1

**FIGURE 2:**
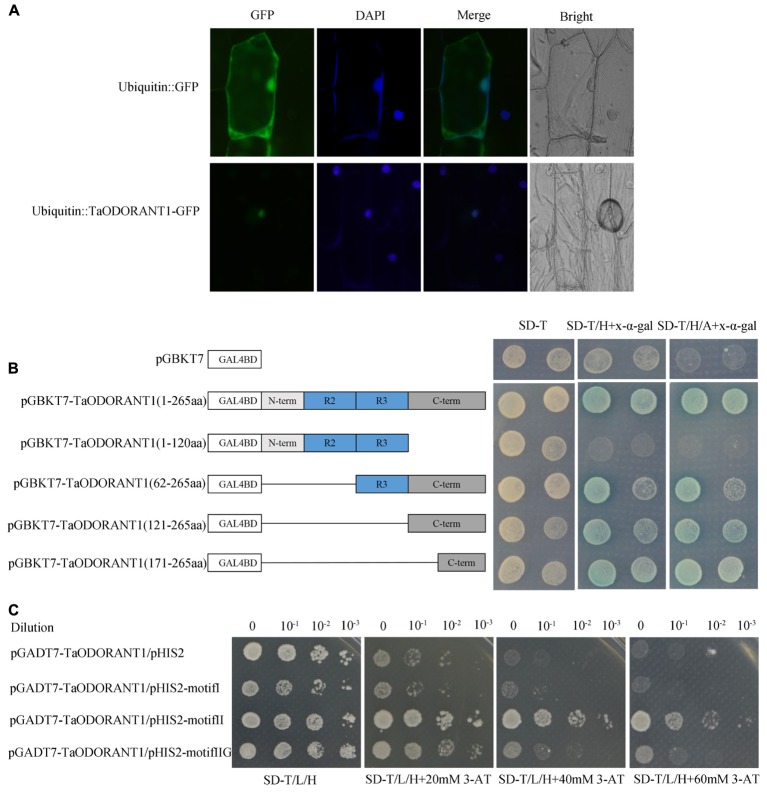
Subcellular localization and transcriptional activity analysis of TaODORANT1. **(A)** Subcellular localization of TaODORANT1. Recombinant vector ubiqutin::TaODORANT1-GFP and control vector ubiquitin::GFP were transformed into onion epidermal cells and observed with fluorescence microscopy, respectively. **(B)** Transactivation activity of TaODORANT1 in yeast. Schematic diagrams illustrate the different portions of TaODORANT1 ORF that were introduced into the pGBKT7. Recombined vectors were transformed into yeast strain AH109, and the transformants were screened by SD/-Trp, SD/-Trp/-His+X-α-gal, SD/-Trp/-His/-Ade+X-α-gal media. **(C)** Binding activity assay of TaODORANT1. Three MYB binding motifs I, II, and IIG were fused with pHIS2 vector, and the *TaODORANT1* ORF was fused with pGADT7 vector. Reconstructed pGADT7 and pHIS2 vectors were co-transformed into yeast strain Y187. Transformants were incubated on SD/-Trp/-Leu/-His media with different concentrations of 3-AT. Three independent biological replicates were performed and produced similar results.

To confirm the localization of TaODORANT1 *in vivo*, a vector that expressed the fused TaODORANT1-GFP protein under the control of a maize *ubiquitin* promoter was constructed. Transient expression of the fused protein gene in onion epidermal cells showed that the fluorescence of TaODORANT1-GFP was exclusively localized in the nucleus, whereas that of the control GFP protein was diffused throughout the cell (**Figure 2A**). These results suggested that TaODORANT1 was a nuclear-localized protein.

The transactivation activity of TaODORANT1 was verified with a transactivation assay. To construct GAL4BD-TaODORANT1 recombinant, complete and various truncated *TaODORANT1* ORFs were cloned into pGBKT7 plasmids. The recombinants were transformed into the yeast strain AH109 to examine the transactivation ability of TaODORANT1. All transformants, including the negative control pGBKT7, grew well on the SD/-Trp medium, while just transformants containing the TaODORANT1 C-terminal grew well and turned blue on SD-Trp/His and SD-Trp/His/Ade medium with x-α-galactoside (x-α-gal) (**Figure 2B**). These results indicated that TaODORANT1 had transactivation activity, which can be attributed to the amino acid residues at C-terminal 171 to 265. Furthermore, binding activity analysis showed that the fused protein pGADT7-TaODORANT1 was able to bind to type II but not to type I and IIG MYB binding motifs (**Figure 2C**).

### Expression Pattern Analysis of *TaODORANT1* in Wheat

**FIGURE 3:**
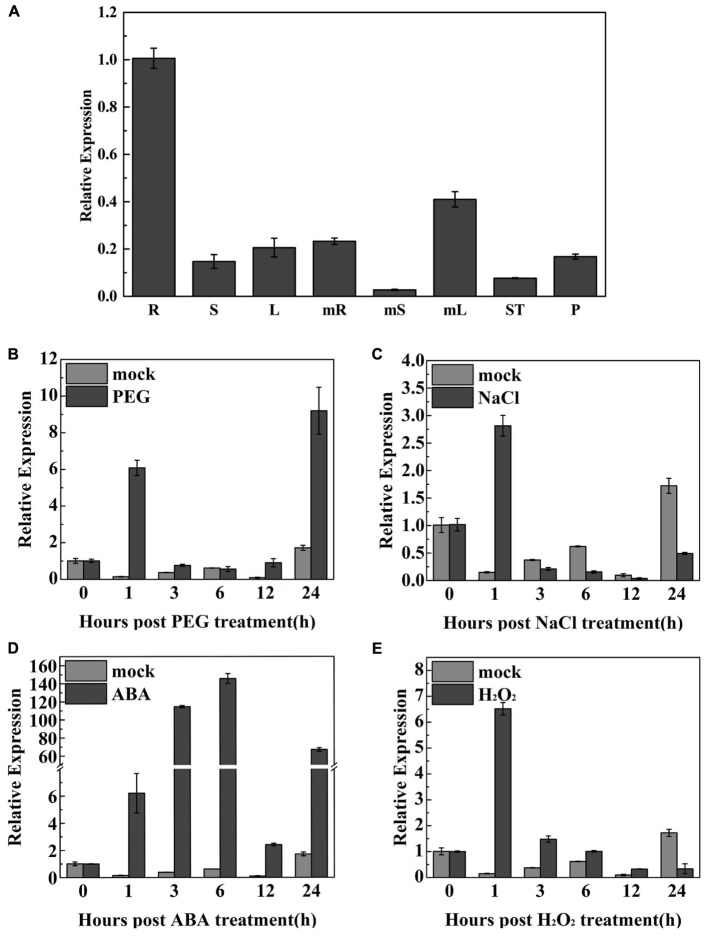
Expression profiles of *TaODORANT1* in wheat. **(A)** Organ-specific expression assay of *TaODORANT1* in wheat (R: seedlings root; S: seedlings stem; L: seedlings leaf; mR: mature root; mS: mature stem; mL: mature leaf; ST: stamen; P: pistil). **(B–E)** Expression patterns of *TaODORANT1* in 14-day-old wheat seedlings after treatment with 20% PEG6000, 200 mM NaCl, 100 μM ABA, and 10 mM H_2_O_2_, respectively. Three independent biological replicates were performed and produced similar results. Vertical bars refer to ±SE (*n* = 3).

qRT-PCR was used to examine *TaODORANT1* expression in various wheat organs at different developmental stages. Results showed that *TaODORANT1* was expressed in all examined organs. The highest transcript levels were observed in the roots of 2-week-old seedlings. The lowest expression levels were observed in mature stems (**Figure 3A**). To gain insight into the function of *TaODORANT1*, its expression levels were measured under various stress treatments. *TaODORANT1* expression rapidly increased to 6.1-fold at 1 h, and then gradually returned to normal levels after 3 h to 12 h of 20% PEG6000 treatment. After 24 h of 20% PEG6000 treatment, *TaODORANT1* expression sharply increased to its highest level of approximately nine-fold (**Figure 3B**). After treatment with 200 mM NaCl, *TaODORANT1* expression increased 2.8-fold at 1 h and then quickly decreased (**Figure 3C**). Given that ABA and H_2_O_2_ are induced and accumulate as signal molecules under drought and salt stresses, *TaODORANT1* expression levels were also examined after ABA and H_2_O_2_ treatments. Under 100 μM ABA treatment, *TaODORANT1* expression levels initially increased, gradually reached peak, and then decreased (**Figure 3D**). Under 10 mM H_2_O_2_ treatment, *TaODORANT1* expression increased (6.8-fold) at 1 h and then gradually decreased to a normal level (**Figure 3E**). These results demonstrated that the expression of *TaODORANT1* in wheat was induced by PEG6000, NaCl, H_2_O_2_, and ABA. To further understand the regulatory mechanism of *TaODORANT1* expression, a 1,800-bp fragment in the promoter region of *TaODORANT1* was cloned and analyzed. Many abiotic stress response elements (MBS, LTR, HSE, ARBE, and ERE) were found in the promoter sequence (Supplementary Table S2). Based on these results, we speculated that TaODORANT1 plays a key role in plant response to adverse environments.

### Ectopic Overexpression of *TaODORANT1* Enhances Drought and Salt Tolerance in Transgenic Tobacco Seedlings

**FIGURE 4:**
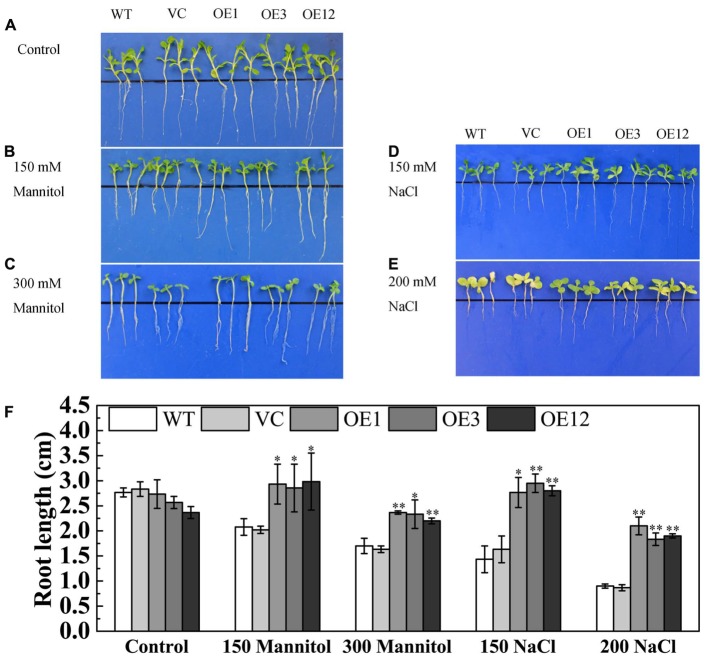
Tolerance analysis of 10-day-old tobacco seedlings. **(A–E)** Phenotype of seedlings after treatment with mannitol or NaCl for 10 days. **(F)** Root length statistics of seedlings after treatment with mannitol or NaCl for 10 days. Three independent biological replicates were performed and produced similar results. Vertical bars refer to ±SE (*n* = 3). Asterisks indicate significant difference between WT and transgenic lines (^∗^*P* < 0.05; ^∗∗^*P* < 0.01).

To further investigate the function of *TaODORANT1* in abiotic stress tolerance, transgenic tobacco plants were generated. Then, the expression levels of transgene were detected by semi-qRT-PCR, and result showed that the three *TaODORANT1-*overexpressing lines OE1, OE3, and OE12 had higher expression levels. The copy number of transgene was detected by Southern blotting, and the result revealed that one (OE3) or two copies (OE1 and OE12) of *TaODORANT1* were integrated into the genome of these three tobacco plants (Supplementary Figure S1). Therefore, these three independent lines were selected to analyze the function of *TaODORANT1* in our study. Under normal conditions, the WT, VC, and OE lines showed similar phenotypes. For the drought/salt stress tolerance assay of *TaODORANT1* overexpressing plants, the 10-day-old post-germination seedlings were vertically grown on 1/2 MS media that contained 150/300 mM mannitol or 150/200 mM NaCl for 10 days. The transgenic and control lines showed no difference in growth on 1/2 MS plates. By contrast, the growth of the control lines on stress media was more inhibited than that of the OE lines (**Figures 4A–E**). Statistical analysis of root length also showed that the overexpression lines grew better than the control lines under NaCl and mannitol treatments (**Figure 4F**). These results indicated that *TaODORANT1* overexpression enhanced tobacco seedlings tolerance to drought and salt stresses.

### Drought Tolerance Assay of *TaODORANT1* Overexpression Tobacco Plants

**FIGURE 5:**
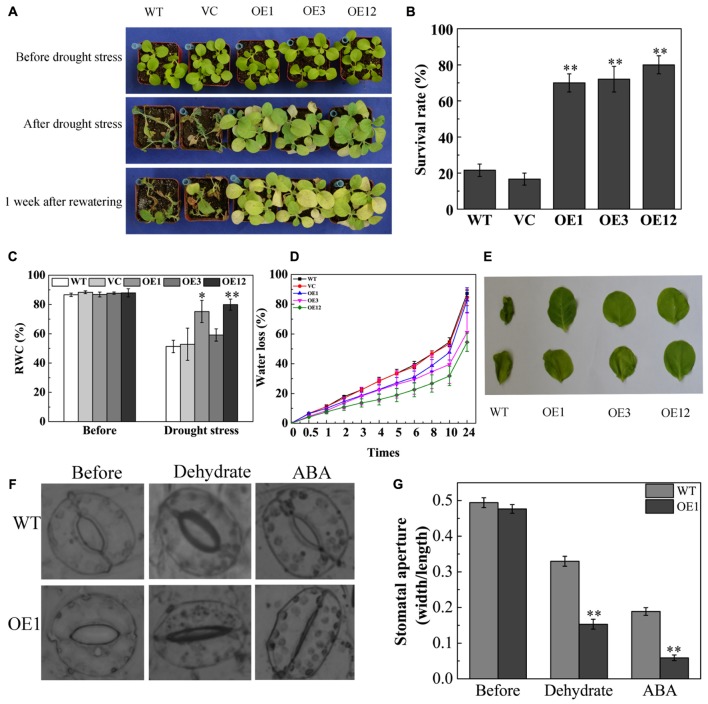
Drought tolerance of *TaODORANT1* overexpressed tobacco plants. **(A)** Phenotype of WT, VC, and OE lines (OE1, OE3, and OE12) after drought treatment. **(B)** Survival rate statistics after re-watering for 1 week. **(C)** RWC of leaves after drought stress. **(D)** Water loss rate of detached leaves. **(E)** Phenotype of detached leaves after 24 h dehydration. **(F)** Stomatal aperture after dehydration or ABA treatment. **(G)** Width/length ratio of stomata. Three independent biological replicates were performed and produced similar results. Vertical bars refer to ±SE (*n* = 3). Asterisks indicate significant difference between the WT and the transgenic lines (^∗^*P* < 0.05; ^∗∗^*P* < 0.01).

To further investigate drought tolerance of *TaODORANT1* overexpressing plants, water was withheld from 4-week-old plants in soil for 27 days. The WT and VC lines became seriously chlorotic at an early stage, wilted, and even died. By contrast, the transgenic lines OE1, OE3, and OE12 showed only chlorosis and few deaths (**Figure 5A**). After re-watering for 1 week, the survival rate of the transgenic plants was 60–80%, which was obviously higher than those of WT and VC (<20%) (**Figure 5B**). The RWC of leaves was measured as an important parameter of drought tolerance. The OE1 and OE12 lines exhibited higher RWC (70–80%) than that of the control plants (40–45%) (**Figure 5C**). Consistent with the drought tolerance phenotype, the water loss rate of detached leaves from the WT and VC lines was also higher than those from transgenic lines (**Figure 5D**). Moreover, the WT leaves presented serious coiling after 24 h of dehydration (**Figure 5E**). As more than 95% of water loss in plants is *via* transpiration (Schroeder et al., 2001), the status of stomatal closure was observed and the stomatal width:length ratio was measured under dehydration or exogenous ABA treatments. Result showed that the stomatal aperture of OE1 was smaller than that of the WT (**Figures 5F,G**). These results demonstrated that TaODORANT1 positively regulated drought stress response.

### Salt Stress Tolerance Assay of *TaODORANT1*-Overexpressing Tobacco Plants

**FIGURE 6:**
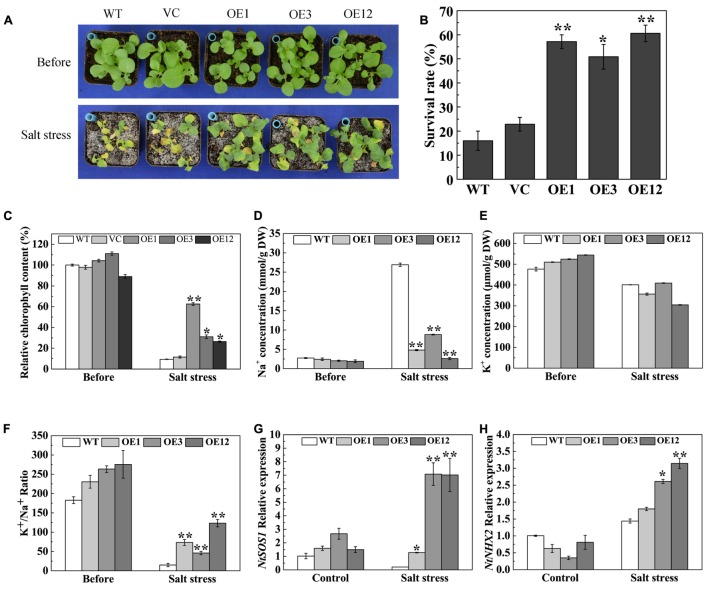
Salt tolerance of *TaODORANT1* overexpressed tobacco plants. **(A)** Phenotype of WT, VC and OE lines (OE1, OE3, and OE12) after salt treatment. **(B)** Survival rate statistical analysis. **(C)** Chlorophyll content in leaves. **(D,E)** Na^+^ and K^+^ concentrations in leaves. **(F)** K^+^/Na^+^ ratio. **(G,H)** Expression levels of ion transporter genes. Three independent biological replicates were performed and produced similar results. Vertical bars refer to ±SE (*n* = 3). Asterisks indicate significant difference between the WT and the transgenic lines (^∗^*P* < 0.05; ^∗∗^*P* < 0.01).

To analyze the salt stress tolerance of *TaODORANT1* overexpressing plants, 3-week-old plants were irrigated with 500 mM NaCl solution for 19 days. The WT and VC lines exhibited serious chlorosis and death, whereas the transgenic plants remained green (**Figure 6A**) and showed a higher survival rate (50–60%) than WT and VC (**Figure 6B**). The reduced chlorophyll content of the leaves of WT and VC plants also reflected this observed phenotype (**Figure 6C**). To further understand the mechanism underlying the enhanced salt tolerance of *TaODORANT1* overexpressing plants, Na^+^ and K^+^ accumulation in leaves was detected after salt stress. No significant difference of K^+^ concentration was observed between WT and OE lines (**Figure 6E**). However, Na^+^ concentration was significantly lower in OE lines than WT (**Figure 6D**). Therefore, the K^+^/Na^+^ ratios in OE lines were higher than that in WT after salt stress (**Figure 6F**). The expression levels of two representative ion transporter genes were examined by qRT-PCR. The expression levels of *NtSOS1* and *NtNHX2* were higher in transgenic plants (OE3 and OE12) than those in WT plants (**Figures 6G,H**). These results suggested that TaODORANT1 regulates the expression of Na^+^ and K^+^ transporter genes, thereby reducing Na^+^ accumulation in photosynthetic tissue and improving salt stress tolerance of transgenic plants.

### Oxidative Damage Assay of *TaODORANT1* Overexpression Tobacco Plants under Drought and Salt Stresses

**FIGURE 7:**
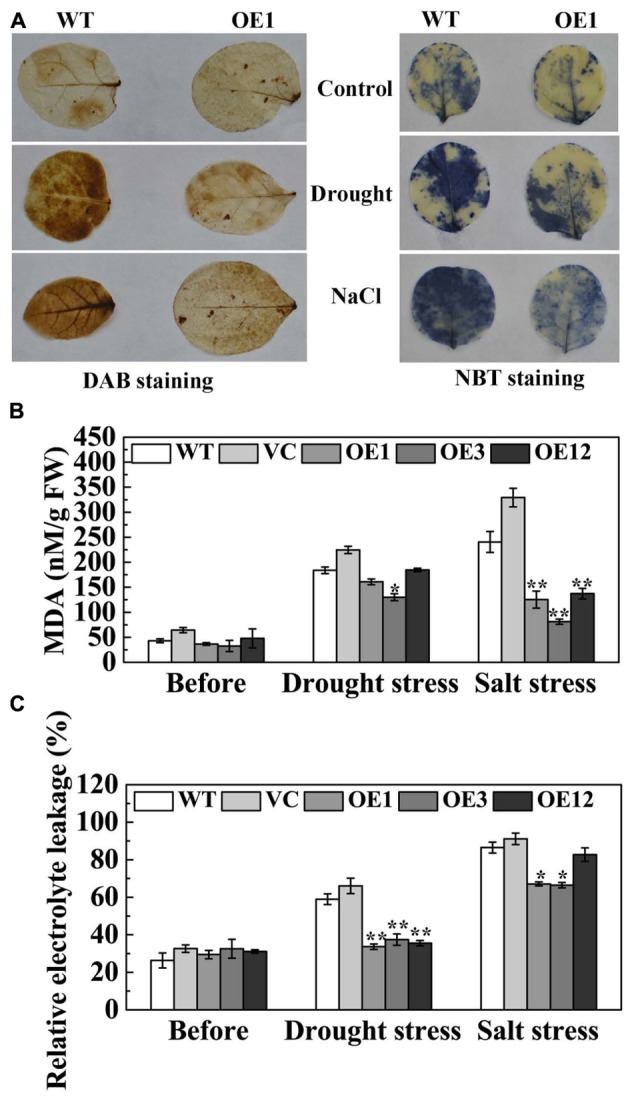
Oxidative damage of transgenic tobacco plants after drought/salt stress. **(A)** Histochemical detection of H_2_O_2_ and O_2_^-^ by DAB staining and NBT staining. **(B)** MDA content and **(C)** IL under normal and drought/salt stress. Three independent biological replicates were performed and produced similar results. Vertical bars refer to ±SE (*n* = 3). Asterisks indicate significant difference between the WT and the transgenic lines (^∗^*P* < 0.05; ^∗∗^*P* < 0.01).

Drought and salt stresses disrupt cellular homeostasis and increase the accumulation of reactive oxygen species (ROS), especially O_2_^-^ and H_2_O_2_ (Polle, 2001). The histochemical detection of H_2_O_2_ and O_2_^-^ in leaves was accomplished by DAB and NBT staining. Under normal conditions, H_2_O_2_ and O_2_^-^ contents were relatively lower and not significantly different between WT and OE1 lines. After drought or NaCl treatment, the staining of WT and OE1 leaves was deeper than that seen in the control. However, the staining of OE1 leaves was lighter than the WT (**Figure 7A**), which indicated that transgenic plants accumulated less H_2_O_2_ and O_2_^-^. Under normal conditions, IL and MDA content in the leaves of the WT, VC, and OE lines were not significantly different. After drought stress, the accumulation of MDA in OE3 was significantly less than WT and VC. After salt stress, the accumulation of MDA in transgenic plants (OE1, OE3, and OE12) was significantly lower when compared with the WT and VC. Moreover, the WT and VC leaves presented severe IL compared with transgenic plants after drought and salt stresses (**Figures 7B,C**). These results indicated that *TaODORANT1* overexpression decreased the oxidative damage caused by drought and salt stresses.

### Overexpression of *TaODORANT1* Regulates the Expression Levels of Antioxidation-Related Genes and Improves the Activity of Antioxidative Enzymes under Drought and Salt Stresses

**FIGURE 8:**
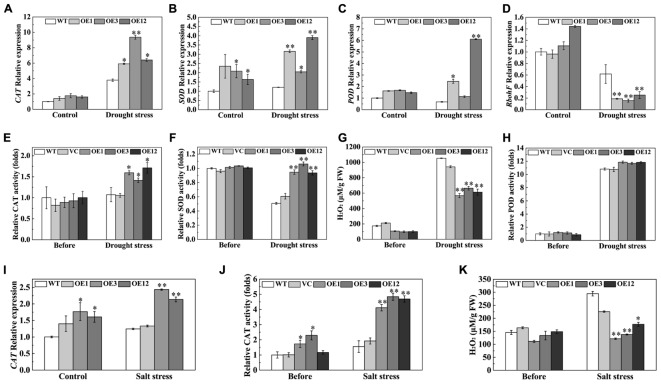
Overexpression of *TaODORANT1* in tobacco elevated antioxidant enzymes gene expression and activities under drought/salt stress. **(A–D)** Expression levels of *CAT*, *SOD*, *POD*, and *RbohF* in tobacco seedlings after 300 mM mannitol treatment for 7 days. **(E–H)** H_2_O_2_ content and activities of CAT, SOD, and POD in plant leaves after drought treatment. **(I)** Expression levels of *CAT* in tobacco seedlings after 150 mM NaCl treatment for 7 days. **(J,K)** CAT activity and H_2_O_2_ content in plant leaves after NaCl treatment. Three independent biological replicates were performed and produced similar results. Vertical bars refer to ±SE (*n* = 3). Asterisks indicate significant difference between the WT and the transgenic lines (^∗^*P* < 0.05; ^∗∗^*P* < 0.01).

An efficient antioxidation system, that alleviates oxidative damage, is required to enhance drought and salt tolerance (Bartels and Sunkar, 2005). The expression levels of *CAT*, *SOD*, and *POD* genes, related to ROS-scavenging enzymes, were examined by qRT-PCR. After drought stress, the transcription levels of *CAT*, *SOD*, and *POD* were higher in OE lines than those in WT (**Figures 8A–C**). On the other hand, expression of the respiratory burst oxidase homolog (*RbohF*), a ROS producer, significantly decreased in the OE lines (**Figure 8D**). H_2_O_2_ content and antioxidative enzyme activities in leaves were detected after drought stress. The results showed that OE lines exhibited higher activities of CAT and SOD, and less H_2_O_2_ accumulation than the WT and VC after drought stress (**Figures 8E–G**). However, POD activity was not significantly different between the OE lines and the control (**Figure 8H**). After salt treatment, the *CAT* transcript levels were higher in the OE3 and OE12 lines than those in the WT (**Figure 8I**). Enhanced CAT activity, which directly decomposes H_2_O_2_, reduced accumulation of H_2_O_2_ in transgenic plants (**Figures 8J,K**). However, SOD and POD activities were not significantly different between the OE lines and the control (Supplementary Figure S2). These results showed that TaODORANT1 enhanced ROS-scavenging ability by activating the activities of different enzymes in response to drought and salt stresses.

### TaODORANT1 Regulates the Expressions of Stress-Related Genes under Drought and Salt Stresses

**FIGURE 9:**
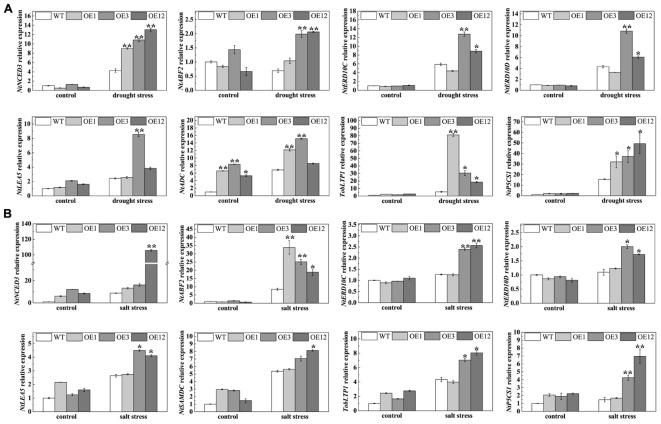
Expression analysis of stress-related genes. **(A,B)** Expression levels of stress-related genes in tobacco seedlings after treatment on 1/2 MS media with 300 mM mannitol or 150 mM NaCl for 1 week. Three independent biological replicates were performed and produced similar results. Vertical bars refer to ±SE (*n* = 3). Asterisks indicate significant difference between the WT and the transgenic lines (^∗^*P* < 0.05; ^∗∗^*P* < 0.01).

To further understand the functional mechanisms of TaODORANT1 at the molecular level, qRT-PCR was used to examine the levels of stress-related genes, including *NtNCED3* (9-*cis*-epoxycarotenoid dioxygenase), *NtERD10C/D* (early responsive to dehydration), *NtABF2* (ABA-responsive element binding), *NtLEA5* (late embryogenesis-abundant protein), *TobLTP1* (lipid-transfer protein), *NtP5CS1* (Δ1-pyrroline-5-carhoxylate synthetase 1), *NtSAMDC* (*S*-adenosyl-L-methionine decarboxylase), and *NtADC* (arginine decarboxylase). Under 300 mM mannitol or 150 mM NaCl treatment, the expression levels of *NtNCED3*, *NtERD10C*, *NtERD10D*, *NtLEA5*, *NtABF2*, *TobLTP1*, *NtP5CS1*, *NtSAMDC*, and *NtADC* were higher in transgenic lines (OE3 and OE12) than those in WT. However, the expression of some related genes in OE1 were not significantly induced after drought and salt stresses (**Figure 9**). These results suggested that *TaODORANT1* overexpression in tobacco plants enhanced the drought and salt tolerance by regulating the expression of stress-related genes.

## Discussion

Biotic and abiotic stresses seriously influence the growth, development, and productivity of crop plants. Therefore, stress tolerance genes should be explored for the genetic improvement of crops. Although many TFs involved in plants abiotic stress have been identified and characterized, few *MYB* genes have so far been characterized in wheat. Furthermore, the mechanisms through which *MYB* genes enhance plant drought and salt stress tolerance remain unclear. In our study, a R2R3-type MYB gene *TaODORANT1* was cloned. Sequence alignment showed that TaODORANT1 had 98% identity with protein ODORANT1 (EMT12444) from *A. tauschii* which has yet to be functionally characterized. Promoter element analysis suggested that *TaODORANT1* may be involved in responses to abiotic stress in plants. For example, the MBS *cis-*element is observed in the promoter of *RD29*, which has been identified to be a typical desiccation-responsive gene (Yamaguchi-Shinozaki and Shinozaki, 1993). The up-regulated expression of *TaODORANT1* in wheat after PEG6000, NaCl, ABA, and H_2_O_2_ treatments, also suggested that this gene was involved in abiotic stress in plants (**Figure 3B**). Tobacco plants that over-expressed *TaODORANT1* had enhanced drought and salt stress tolerance (**Figures 4**–**6**), which is similar to the function of other *MYB* genes in wheat (Mao et al., 2011; He et al., 2012; Qin et al., 2012; Zhang L.C. et al., 2012).

Under drought stress, stomatal closure in *TaODORANT1* transgenic plants was more sensitive to dehydration and ABA treatments than that in WT plants. Moreover, *TaODORANT1*-overexpressing plants had higher RWC and lower water loss in leaves (**Figures 5C–G**). Chen et al. (2015) also observed that GbMYB5 decreased stomatal aperture and reduced water loss rate in transgenic tobacco under drought stress. A reduced rate of water loss is crucial for plant survival under drought conditions. Transpiration, the process by which water is transported to leaves and released vapor through stomatal pores, is the principal pathway leading to water loss. Acting as a signal molecule, ABA perceives stresses and triggers changes in guard cells that stimulate stomatal closure, thus reducing transpiration (Raghavendra et al., 2010). These results agreed with previous reports for TaMYB3R1, AtMYB44, and AtMYB60, all of which have been showed to decrease the stomatal aperture in response to drought/osmotic stress (Cominelli et al., 2005; Jung et al., 2008; Cai et al., 2015). These results suggested that TaODORANT1 might be involved in ABA-mediated stomatal closure in adaptation to adverse environmental conditions.

Under salt stress, a higher Na^+^ concentration disrupts ion homeostasis, which contributes to Na^+^ toxicity and disrupts K^+^ homeostasis in the cytosol. Na^+^ concentration in the cytoplasm is maintained by restricting Na^+^ uptake, promoting Na^+^ extrusion across the plasma membrane, and sequestering Na^+^ in vacuoles under a high-salt environment (Bartels and Sunkar, 2005). The gene *SOS1* encodes a plasma membrane Na^+^/H^+^ antiporter that transports Na^+^ out of cells and partitions Na^+^ between plant organs. *AtSOS1* overexpression improves salt tolerance in tobacco by maintaining higher K^+^/Na^+^ ratios (Yue et al., 2012). The SlSOS1 antiporter not only maintains ion homeostasis, but also partitions Na^+^ to stems to reduce Na^+^ contents in photosynthetic tissues (Olias et al., 2009). Coincidentally, the SbSOS1 antiporter partitions Na^+^ to stem and xylem tissues to reduce Na^+^ in leaves and roots, thereby enhancing the salt stress tolerance of tobacco plants (Yadav et al., 2012). The gene *NHX2* encodes a K^+^ and Na^+^/H^+^ antiporter, which is localized in the tonoplast. AtNHX2, as a salt tolerance determinant, functions in compartmentalizing Na^+^ in the vacuole (Yokoi et al., 2002). In the present study, compared with WT, *TaODORANT1-*overexpressing lines exhibited elevated *NtSOS1* and *NtNHX2* expression levels and increased K^+^/Na^+^ ratio in leaves after salt stress (**Figures 6C–F**). These results demonstrated that TaODORANT1 increased the K^+^/Na^+^ ratio in leaves by regulating ion transporters to enhance tolerance to salt stress.

Drought and salt stresses can lead to redundant ROS accumulation, resulting in severe oxidative damage in plants. ROS accumulation causes peroxidation of membrane lipids, which produces a mass of degradation products, such as MDA (Moore and Roberts, 1998). ROS accumulation is also responsible for severe electrolyte leakage. Demidchik et al. (2014) indicated that ROS-activated outwardly rectifying K^+^ channels resulted in K^+^ release, a major component of electrolyte leakage, from plant cells. To scavenge ROS accumulation, plants form an enzymatic system that consists of ROS-scavenging enzymes, such as SOD, CAT, and POD. Under drought stress, the expression levels and the enzyme activities of SOD and CAT were higher in transgenic plants than those in control plants. Meanwhile, the expression levels of *RbohF*, which encodes a ROS producer, decreased in OE plants (**Figures 8A–F**). Under salt stress, both expression level and enzyme activity of CAT were higher in transgenic plants (**Figures 8I,J**). However, POD activity under drought stress as well as POD and SOD activities under salt stress were not significantly increased compared with the WT (**Figure 8H** and Supplementary Figure S2). The results indicated that the antioxidative system is complicated. Antioxidant capacity is very much dependent on the severity and type of stress. Under different stress treatments, various plants may favor one mechanism to preferentially scavenge redundant ROS (Dat et al., 2000). The alleviative oxidative damage in transgenic tobacco plants was corroborated by decreased IL, and H_2_O_2_ and MDA contents (**Figures 7**, **8G,K**). These results demonstrated that TaODORANT1 activated the ROS-scavenging system to relieve oxidative damage and enhance drought and salt tolerance.

To further understand the mechanism of enhanced stress tolerance in *TaODORANT1*-overexpressing transgenic plants, the transcript levels of several stress-related genes were examined. *NCED* encodes the rate-limiting enzyme of ABA biosynthesis and accumulation (Qin and Zeevaart, 1999). *ABF2* encodes a bZIP TF that specifically binds to the ABRE *cis*-element; this TF is involved in the ABA signal pathway, which responds to abiotic stress (Yoshida et al., 2010). *LEA5* and *ERD10 (C/D)* encode group 2 and group 5 LEA proteins, respectively; these proteins maintain the structural stability of proteins and membranes (Amara et al., 2012). The lipid-transfer protein gene *LTP1* is induced by ABA, cold, drought, and salt stress (Hu et al., 2013). The enzyme genes *NtP5CS1*, *NtSAMDC*, and *NtADC* participate in biosynthesis of the osmoprotectants proline and polyamine, which function in resisting adverse environments by adjusting osmotic balance and protecting plasma membrane integrity (Bartels and Sunkar, 2005). All the above-mentioned genes were up-regulated in *TaODORANT1*-overexpressing tobacco plants under drought and salt stresses (**Figure 9**). These results demonstrated that TaODORANT1 improves the transcription of stress-related genes in response to drought and salt stresses. The result agreed with previous studies that the stress tolerance of transgenic plants was enhanced by up-regulating the expression of stress related genes (Hu et al., 2013; Wang et al., 2015).

In summary, TaODORANT1 is a MYB transcriptional activator that is induced by PEG6000, NaCl, ABA, and H_2_O_2_ in wheat. *TaODORANT1* overexpression in tobacco enhances drought and salt tolerance by increasing RWC and decreasing water loss, as well as reducing H_2_O_2_, MDA, and Na^+^ accumulation. Moreover, *TaODORANT1* overexpression improves the activity of antioxidant enzymes and the expression of stress-related genes. Future study of the direct downstream gene or protein targets of TaODORANT1 will contribute to further elucidation of the mechanisms of TaODORANT1-mediated stress tolerance.

## Author Contributions

GH, GY, JC, and QW designed the experiments and wrote the paper. QW performed all experiments and analyzed the data. QL helped to perform genetic transformation and partial data analysis. RW, FZ, YH, YZ, DQ, and KL participated in physiological assays and gene expression assays. All authors read and approved the manuscript.

## Conflict of Interest Statement

The authors declare that the research was conducted in the absence of any commercial or financial relationships that could be construed as a potential conflict of interest.

## Footnotes

1 http://www.arabidopsis.org

2 http://rice.plantbiology.msu.edu

3 http://www.plexdb.org

4 http://plants.ensembl.org/index.html

5 http://www.wheatgenome.org

6 http://bioinformatics.psb.ugent.be/webtools/plantcare/html

7 https://blast.ncbi.nlm.nih.gov/Blast.cgi

8 https://www.ncbi.nlm.nih.gov/unigene

## References

[B1] Agarwal M. Hao Y. J. Kapoor A. Dong C. H. Fujii H. Zheng X. W. (2006). A R2R3 type MYB transcription factor is involved in the cold regulation of CBF genes and in acquired freezing tolerance. *J. Biol. Chem.* 281 37636–37645. 10.1074/jbc.M605895200 17015446

[B2] Amara I. Odena A. Oliveira E. Moreno A. Masmoudi K. Pages M. (2012). Insights into Maize LEA proteins: from proteomics to functional approaches. *Plant Cell Physiol.* 53 312–329. 10.1093/pcp/pcr183 22199372

[B3] Arnon D. I. (1949). Copper enzymes in isolated chloroplasts. Polyphenoloxidase in beta vulgaris. *Plant Physiol.* 24 1–15. 16654194 10.1104/pp.24.1.1 PMC437905

[B4] Bartels D. Sunkar R. (2005). Drought and salt tolerance in plants. *Crit. Rev. Plant. Sci.* 24 23–58. 10.1080/07352680590910410

[B5] Cai H. S. Tian S. Dong H. S. Guo C. H. (2015). Pleiotropic effects of TaMYB3R1 on plant development and response to osmotic stress in transgenic *Arabidopsis*. *Gene* 558 227–234. 10.1016/j.gene.2014.12.066 25560188

[B6] Chen L. G. Song Y. Li S. J. Zhang L. P. Zou C. S. Yu D. Q. (2012). The role of WRKY transcription factors in plant abiotic stresses. *Biochim. Biophys. Acta* 1819 120–128. 10.1016/j.bbagrm.2011.09.002 21964328

[B7] Chen T. Z. Li W. J. Hu X. H. Guo J. R. Liu A. M. Zhang B. L. (2015). A cotton MYB transcription factor, GbMYB5, is positively involved in plant adaptive response to drought stress. *Plant Cell Physiol.* 56 917–929. 10.1093/pcp/pcv019 25657343

[B8] Chen Y. Chen Z. L. Kang J. Q. Kang D. M. Gu H. Y. Qin G. J. (2013). AtMYB14 regulates cold tolerance in arabidopsis. *Plant Mol. Biol. Rep.* 31 87–97. 10.1007/s11105-012-0481-z 24415840 PMC3881570

[B9] Choulet F. Alberti A. Theil S. Glover N. Barbe V. Daron J. (2014). Structural and functional partitioning of bread wheat chromosome 3B. *Science* 345:1249721 10.1126/Science.1249721 25035497

[B10] Cominelli E. Galbiati M. Vavasseur A. Conti L. Sala T. Vuylsteke M. (2005). A guard-cell-specific MYB transcription factor regulates stomatal movements and plant drought tolerance. *Curr. Biol.* 15 1196–1200. 10.1016/j.cub.2005.05.048 16005291

[B11] Cui M. H. Yoo K. S. Hyoung S. Nguyen H. T. K. Kim Y. Y. Kim H. J. (2013). An Arabidopsis R2R3-MYB transcription factor, AtMYB20, negatively regulates type 2C serine/threonine protein phosphatases to enhance salt tolerance. *FEBS Lett.* 587 1773–1778. 10.1016/j.febslet.2013.04.028 23660402

[B12] Dai X. Y. Xu Y. Y. Ma Q. B. Xu W. Y. Wang T. Xue Y. B. (2007). Overexpression of an R1R2R3 MYB gene, *OsMYB3R-2*, increases tolerance to freezing, drought, and salt stress in transgenic Arabidopsis. *Plant Physiol.* 143 1739–1751. 10.1104/pp.106.094532 17293435 PMC1851822

[B13] Dat J. Vandenabeele S. Vranova E. Van Montagu M. Inze D. Van Breusegem F. (2000). Dual action of the active oxygen species during plant stress responses. *Cell. Mol. Life Sci.* 57 779–795. 10.1007/s000180050041 10892343 PMC11147059

[B14] Demidchik V. Straltsova D. Medvedev S. S. Pozhvanov G. A. Sokolik A. Yurin V. (2014). Stress-induced electrolyte leakage: the role of K+-permeable channels and involvement in programmed cell death and metabolic adjustment. *J. Exp. Bot.* 65 1259–1270. 10.1093/jxb/eru004 24520019

[B15] Dubos C. Stracke R. Grotewold E. Weisshaar B. Martin C. Lepiniec L. (2010). MYB transcription factors in *Arabidopsis*. *Trends Plant Sci.* 15 573–581. 10.1016/j.tplants.2010.06.005 20674465

[B16] Gao S. Zhang Y. L. Yang L. Song J. B. Yang Z. M. (2014). AtMYB20 is negatively involved in plant adaptive response to drought stress. *Plant Soil* 376 433–443. 10.1007/s11104-013-1992-6

[B17] Guo L. Yang H. B. Zhang X. Y. Yang S. H. (2013). Lipid transfer protein 3 as a target of MYB96 mediates freezing and drought stress in *Arabidopsis*. *J. Exp. Bot.* 64 1755–1767. 10.1093/jxb/ert040 23404903 PMC3617838

[B18] He Y. Li W. Lv J. Jia Y. Wang M. Xia G. (2012). Ectopic expression of a wheat MYB transcription factor gene, *TaMYB73*, improves salinity stress tolerance in *Arabidopsis thaliana*. *J. Exp. Bot.* 63 1511–1522. 10.1093/jxb/err389 22140235

[B19] Horsch R. B. Fry J. E. Hoffmann N. L. Eichholtz D. Rogers S. C. Fraley R. T. (1985). A simple and general method for transferring genes into plants. *Science* 227 1229–1231. 10.1126/science.227.4691.1229 17757866

[B20] Hu W. Huang C. Deng X. M. Zhou S. Y. Chen L. H. Li Y. (2013). *TaASR1*, a transcription factor gene in wheat, confers drought stress tolerance in transgenic tobacco. *Plant Cell Environ.* 36 1449–1464. 10.1111/pce.12074 23356734

[B21] Huang P. Chen H. Mu R. Yuan X. Zhang H. S. Huang J. (2015). *OsMYB511* encodes a MYB domain transcription activator early regulated by abiotic stress in rice. *Genet Mol. Res.* 14 9506–9517. 10.4238/2015.August.14.14 26345884

[B22] Huang Q. Wang Y. Li B. Chang J. L. Chen M. J. Li K. X. (2015). TaNAC29, a NAC transcription factor from wheat, enhances salt and drought tolerance in transgenic *Arabidopsis*. *BMC Plant Biol.* 15:268 10.1186/S12870-015-0644-9 PMC4632686 26536863

[B23] Jia J. Z. Zhao S. C. Kong X. Y. Li Y. R. Zhao G. Y. He W. M. (2013). *Aegilops tauschii* draft genome sequence reveals a gene repertoire for wheat adaptation. *Nature* 496 91–95. 10.1038/nature12028 23535592

[B24] Jung C. Seo J. S. Han S. W. Koo Y. J. Kim C. H. Song S. I. (2008). Overexpression of *AtMYB44* enhances stomatal closure to confer abiotic stress tolerance in transgenic Arabidopsis. *Plant Physiol.* 146 623–635. 10.1104/pp.107.110981 18162593 PMC2245844

[B25] Katiyar A. Smita S. Lenka S. K. Rajwanshi R. Chinnusamy V. Bansal K. C. (2012). Genome-wide classification and expression analysis of MYB transcription factor families in rice and Arabidopsis. *BMC Genomics* 13:544 10.1186/1471-2164-13-544 PMC3542171 23050870

[B26] Lee T. G. Jang C. S. Kim J. Y. Kim D. S. Park J. H. Kim D. Y. (2006). A Myb transcription factor (TaMyb1) from wheat roots is expressed during hypoxia: roles in response to the oxygen concentration in root environment and abiotic stresses. *Physiol. Plant.* 129 375–385. 10.1111/j.1399-3054.2006.00828.x

[B27] Ling H. Q. Zhao S. C. Liu D. C. Wang J. Y. Sun H. Zhang C. (2013). Draft genome of the wheat A-genome progenitor *Triticum urartu*. *Nature* 496 87–90. 10.1038/nature11997 23535596

[B28] Liu H. X. Zhou X. Y. Dong N. Liu X. Zhang H. Y. Zhang Z. Y. (2011). Expression of a wheat MYB gene in transgenic tobacco enhances resistance to *Ralstonia solanacearum*, and to drought and salt stresses. *Funct. Integr. Genomics* 11 431–443. 10.1007/s10142-011-0228-1 21597961

[B29] Livak K. J. Schmittgen T. D. (2001). Analysis of relative gene expression data using real-time quantitative PCR and the 2^-ΔΔC_T_^ method. *Methods* 25 402–408. 10.1006/meth.2001.1262 11846609

[B30] Mao X. Jia D. Li A. Zhang H. Tian S. Zhang X. (2011). Transgenic expression of TaMYB2A confers enhanced tolerance to multiple abiotic stresses in *Arabidopsis*. *Funct. Integr. Genomics* 11 445–465. 10.1007/s10142-011-0218-3 21472467

[B31] Mayer K. F. X. Rogers J. Dolezel J. Pozniak C. Eversole K. Feuillet C. (2014). A chromosome-based draft sequence of the hexaploid bread wheat (*Triticum aestivum*) genome. *Science* 345 1251788 10.1126/Science.1251788 25035500

[B32] Meng X. Yin B. Feng H. L. Zhang S. Liang X. Q. Meng Q. W. (2014). Overexpression of R2R3-MYB gene leads to accumulation of anthocyanin and enhanced resistance to chilling and oxidative stress. *Biol. Plant.* 58 121–130. 10.1007/s10535-013-0376-3

[B33] Moore K. Roberts L. J. II (1998). Measurement of lipid peroxidation. *Free Radic. Res.* 28 659–671. 9736317 10.3109/10715769809065821

[B34] Olias R. Eljakaoui Z. Li J. De Morales P. A. Marin-Manzano M. C. Pardo J. M. (2009). The plasma membrane Na+/H+ antiporter *SOS1* is essential for salt tolerance in tomato and affects the partitioning of Na+ between plant organs. *Plant Cell Environ.* 32 904–916. 10.1111/j.1365-3040.2009.01971.x 19302170

[B35] Pasquali G. Biricolti S. Locatelli F. Baldoni E. Mattana M. (2008). *Osmyb4* expression improves adaptive responses to drought and cold stress in transgenic apples. *Plant Cell Rep.* 27 1677–1686. 10.1007/s00299-008-0587-9 18679687

[B36] Paz-Ares J. Ghosal D. Wienand U. Peterson P. A. Saedler H. (1987). The regulatory c1 locus of *Zea mays* encodes a protein with homology to myb proto-oncogene products and with structural similarities to transcriptional activators. *EMBO J.* 6 3553–3558. 3428265 10.1002/j.1460-2075.1987.tb02684.x PMC553820

[B37] Polle A. (2001). Dissecting the superoxide dismutase-ascorbate-glutathione-pathway in chloroplasts by metabolic modeling. Computer simulations as a step towards flux analysis. *Plant Physiol.* 126 445–462. 11351106 10.1104/pp.126.1.445 PMC102317

[B38] Qin F. Shinozaki K. Yamaguchi-Shinozaki K. (2011). Achievements and challenges in understanding plant abiotic stress responses and tolerance. *Plant Cell Physiol.* 52 1569–1582. 10.1093/pcp/pcr106 21828105

[B39] Qin X. Zeevaart J. A. (1999). The 9-cis-epoxycarotenoid cleavage reaction is the key regulatory step of abscisic acid biosynthesis in water-stressed bean. *Proc. Natl. Acad. Sci. U.S.A.* 96 15354–15361. 10611388 10.1073/pnas.96.26.15354 PMC24823

[B40] Qin Y. Wang M. Tian Y. He W. Han L. Xia G. (2012). Over-expression of *TaMYB33* encoding a novel wheat MYB transcription factor increases salt and drought tolerance in *Arabidopsis*. *Mol. Biol. Rep.* 39 7183–7192. 10.1007/s11033-012-1550-y 22350156

[B41] Raghavendra A. S. Gonugunta V. K. Christmann A. Grill E. (2010). ABA perception and signalling. *Trends Plant Sci.* 15 395–401. 10.1016/j.tplants.2010.04.006 20493758

[B42] Rahaie M. Xue G. P. Naghavi M. R. Alizadeh H. Schenk P. M. (2010). A MYB gene from wheat (*Triticum aestivum* L.) is up-regulated during salt and drought stresses and differentially regulated between salt-tolerant and sensitive genotypes. *Plant Cell Rep.* 29 835–844. 10.1007/s00299-010-0868-y 20490502

[B43] Schroeder J. I. Allen G. J. Hugouvieux V. Kwak J. M. Waner D. (2001). Guard cell signal transduction. *Annu. Rev. Plant Physiol. Plant Mol. Biol.* 52 627–658. 10.1146/annurev.arplant.52.1.627 11337411

[B44] Seo P. J. Xiang F. Qiao M. Park J. Y. Lee Y. N. Kim S. G. (2009). The MYB96 transcription factor mediates abscisic acid signaling during drought stress response in *Arabidopsis*. *Plant Physiol.* 151 275–289. 10.1104/pp.109.144220 19625633 PMC2735973

[B45] Staccy J. Isaac P. G. (1994). Isolation of DNA from plants. *Methods Mol. Biol.* 28 9–15. 10.1385/0-89603-254-X:9 8118521

[B46] Tamura K. Peterson D. Peterson N. Stecher G. Nei M. Kumar S. (2011). MEGA5: molecular evolutionary genetics analysis using maximum likelihood, evolutionary distance, and maximum parsimony methods. *Mol. Biol. Evol.* 28 2731–2739. 10.1093/molbev/msr121 21546353 PMC3203626

[B47] Wang X. Zeng J. Li Y. Rong X. Sun J. Sun T. (2015). Expression of *TaWRKY44*, a wheat WRKY gene, in transgenic tobacco confers multiple abiotic stress tolerances. *Front. Plant Sci.* 6:615 10.3389/fpls.2015.00615 PMC4531243 26322057

[B48] Xiong H. Y. Li J. J. Liu P. L. Duan J. Z. Zhao Y. Guo X. (2014). Overexpression of *OsMYB48-1*, a novel MYB-relate transcription factor, enhances drought and salinity tolerance in rice. *PLoS ONE* 9:e92913 10.1371/journal.pone.0092913 PMC3965499 24667379

[B49] Yadav N. S. Shukla P. S. Jha A. Agarwal P. K. Jha B. (2012). The *SbSOS1* gene from the extreme halophyte *Salicornia brachiata* enhances Na+ loading in xylem and confers salt tolerance in transgenic tobacco. *BMC Plant Biol.* 12:188 10.1186/1471-2229-12-188 PMC3548769 23057782

[B50] Yamaguchi-Shinozaki K. Shinozaki K. (1993). Characterization of the expression of a desiccation-responsive *rd29* gene of *Arabidopsis thaliana* and analysis of its promoter in transgenic plants. *Mol. Gen. Genet.* 236 331–340. 8437577 10.1007/BF00277130

[B51] Yan H. R. Jia H. H. Chen X. B. Hao L. L. An H. L. Guo X. Q. (2014). The cotton WRKY transcription factor GhWRKY17 functions in ddrought and salt stress in transgenic *Nicotiana benthamiana* through ABA signaling and the modulation of reactive oxygen species production. *Plant Cell Physiol.* 55 2060–2076. 10.1093/pcp/pcu133 25261532

[B52] Yang A. Dai X. Y. Zhang W. H. (2012). A R2R3-type MYB gene, *OsMYB2*, is involved in salt, cold, and dehydration tolerance in rice. *J. Exp. Bot.* 63 2541–2556. 10.1093/jxb/err431 22301384 PMC3346221

[B53] Yokoi S. Quintero F. J. Cubero B. Ruiz M. T. Bressan R. A. Hasegawa P. M. (2002). Differential expression and function of *Arabidopsis thaliana* NHX Na+/H+ antiporters in the salt stress response. *Plant J.* 30 529–539. 10.1046/j.1365-313X.2002.01309.x 12047628

[B54] Yoshida T. Fujita Y. Sayama H. Kidokoro S. Maruyama K. Mizoi J. (2010). AREB1, AREB2, and ABF3 are master transcription factors that cooperatively regulate ABRE-dependent ABA signaling involved in drought stress tolerance and require ABA for full activation. *Plant J.* 61 672–685. 10.1111/j.1365-313X.2009.04092.x 19947981

[B55] Yue Y. Zhang M. Zhang J. Duan L. Li Z. (2012). SOS1 gene overexpression increased salt tolerance in transgenic tobacco by maintaining a higher K(+)/Na(+) ratio. *J. Plant Physiol.* 169 255–261. 10.1016/j.jplph.2011.10.007 22115741

[B56] Zhang L. Liu G. Zhao G. Xia C. Jia J. Liu X. (2014). Characterization of a wheat R2R3-MYB transcription factor gene, *TaMYB19*, involved in enhanced abiotic stresses in *Arabidopsis*. *Plant Cell Physiol.* 55 1802–1812. 10.1093/pcp/pcu109 25146486

[B57] Zhang L. C. Zhao G. Y. Xia C. Jia J. Z. Liu X. Kong X. Y. (2012). Overexpression of a wheat MYB transcription factor gene, *TaMYB56-B*, enhances tolerances to freezing and salt stresses in transgenic Arabidopsis. *Gene* 505 100–107. 10.1016/j.gene.2012.05.033 22634104

[B58] Zhang Z. Y. Liu X. Wang X. D. Zhou M. P. Zhou X. Y. Ye X. G. (2012). An R2R3 MYB transcription factor in wheat, TaPIMP1, mediates host resistance to *Bipolaris sorokiniana* and drought stresses through regulation of defense- and stress-related genes. *New Phytol.* 196 1155–1170. 10.1111/j.1469-8137.2012.04353.x 23046089

[B59] Zhu N. Cheng S. Liu X. Du H. Dai M. Zhou D. X. (2015). The R2R3-type MYB gene *OsMYB91* has a function in coordinating plant growth and salt stress tolerance in rice. *Plant Sci.* 236 146–156. 10.1016/j.plantsci.2015.03.023 26025528

