# Supplementary material for: A Wheat R2R3-type MYB Transcription Factor TaODORANT1 Positively Regulates Drought and Salt Stress Responses in Transgenic Tobacco Plants
Source: Front Plant Sci. 2017 Aug 8;8:1374. doi: 10.3389/fpls.2017.01374 (PMC5550715; doi:10.3389/fpls.2017.01374)
Supplement: Supplementary file 4 [file Image_1.PDF]

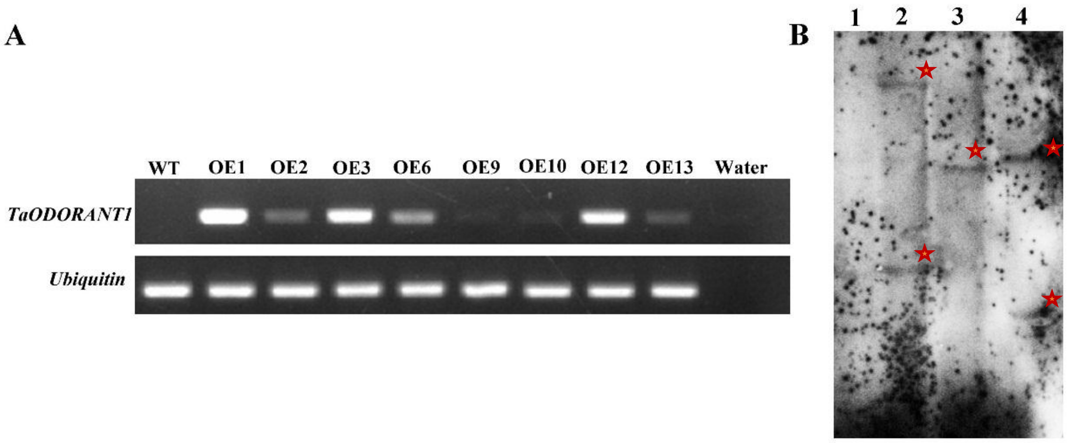

Supplementary Figure S1. Semi-qRT-PCR and Southern blotting analysis of the transgenic tobacco plants. A: The expression levels of *TaODORANT1* in eight independent transgenic tobacco lines. B: Southern blotting analysis of the transgenic tobacco plants. Lane 1: Genomic DNA from WT line digested with *Hin*dIII. Lane 2-4: Genomic DNA from transgenic tobacco plants OE1, OE3, and OE12, respectively, digested with *Hin*dIII.
