# Supplementary material for: A Wheat R2R3-type MYB Transcription Factor TaODORANT1 Positively Regulates Drought and Salt Stress Responses in Transgenic Tobacco Plants
Source: Front Plant Sci. 2017 Aug 8;8:1374. doi: 10.3389/fpls.2017.01374 (PMC5550715; doi:10.3389/fpls.2017.01374)
Supplement: Supplementary file 5 [file Image_2.PDF]

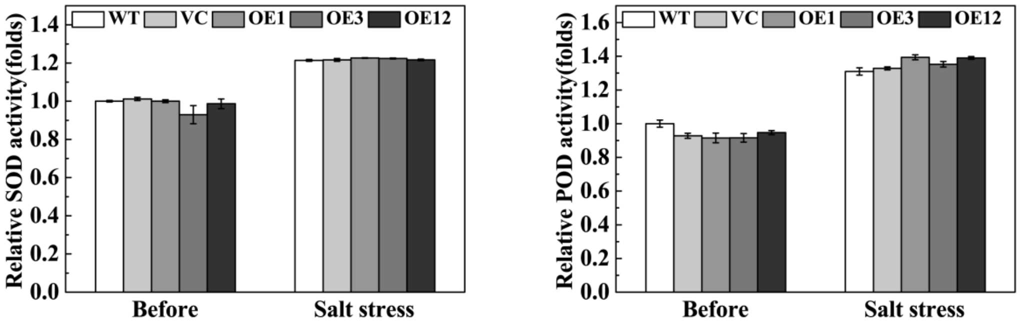

Supplementary Figure S2. The enzyme activities of SOD and POD in leaves of transgenic tobacco after salt stress.
