# Supplementary material for: A Wheat R2R3-type MYB Transcription Factor TaODORANT1 Positively Regulates Drought and Salt Stress Responses in Transgenic Tobacco Plants
Source: Front Plant Sci. 2017 Aug 8;8:1374. doi: 10.3389/fpls.2017.01374 (PMC5550715; doi:10.3389/fpls.2017.01374)
Supplement: Supplementary file 6 [file Image_3.PDF]

A

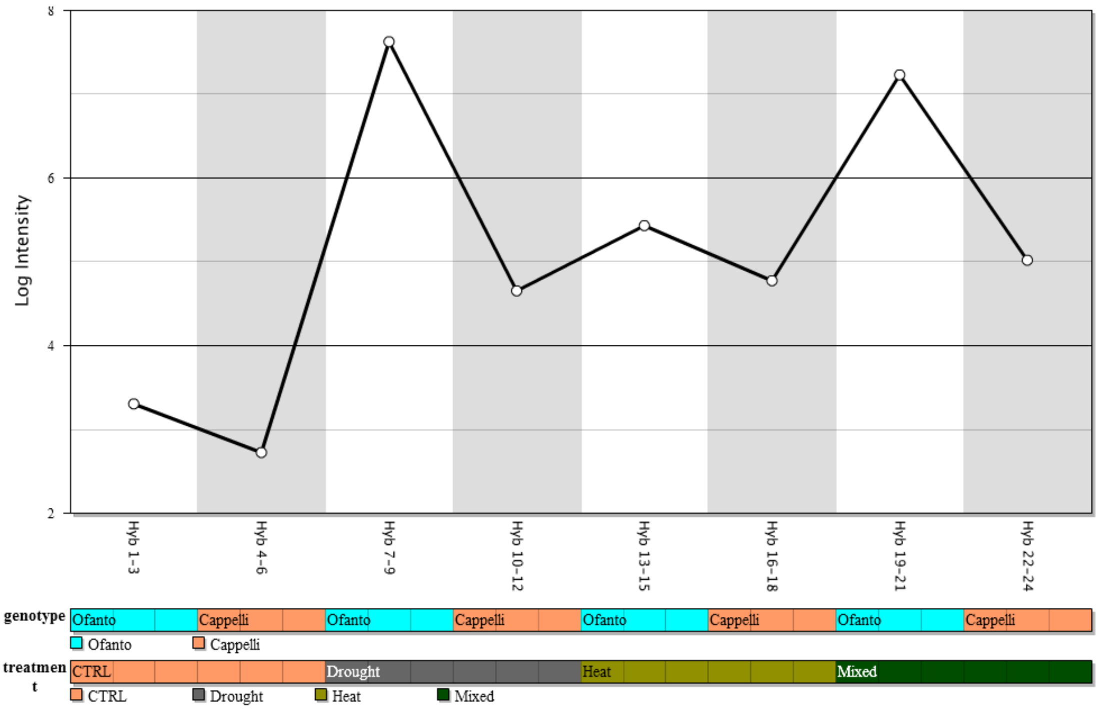

Log Intensity
8
6
4
2
Hyb 1-3
Hyb 4-6
Hyb 7-9
Hyb 10-12
Hyb 13-15
Hyb 16-18
Hyb 19-21
Hyb 22-24
genotype
Ofanto
Cappelli
Ofanto
Cappelli
Ofanto
Cappelli
Ofanto
Cappelli
Ofanto
Cappelli
treatment
CTRL
Drought
Heat
Mixed
CTRL
Drought
Heat
Mixed

B

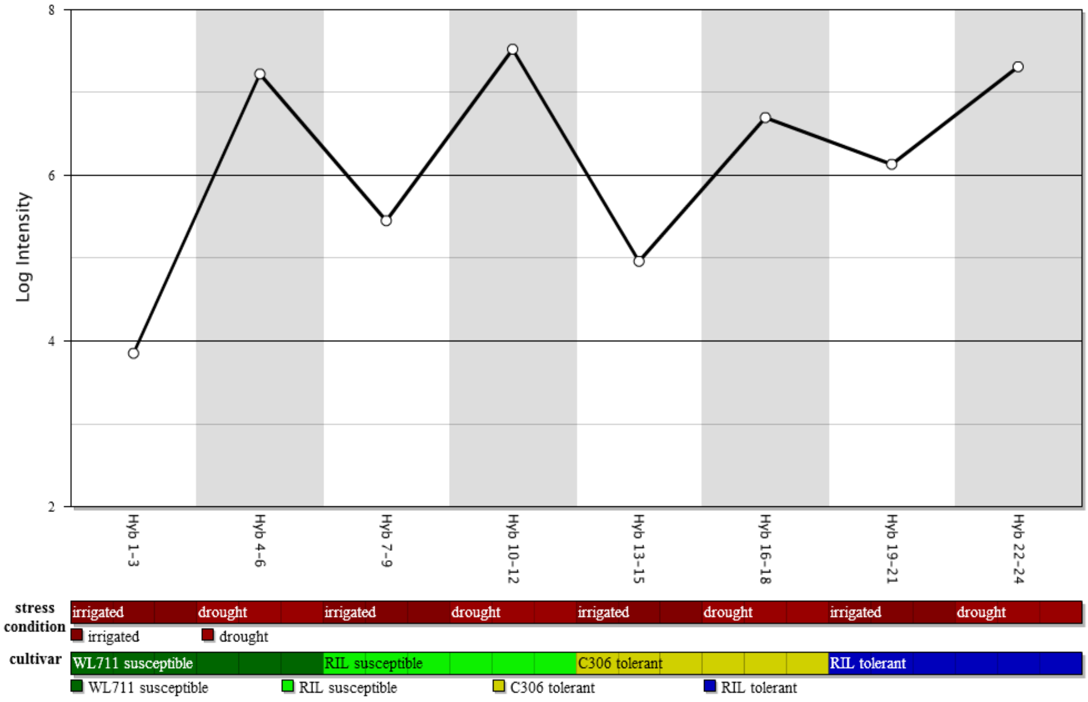

Log Intensity
8
6
4
2
Hyb 1-3
Hyb 4-6
Hyb 7-9
Hyb 10-12
Hyb 13-15
Hyb 16-18
Hyb 19-21
Hyb 22-24
stress condition
irrigated
drought
irrigated
drought
irrigated
drought
irrigated
drought
irrigated
drought
cultivar
WL711 susceptible
RIL susceptible
C306 tolerant
RIL tolerant
WL711 susceptible
RIL susceptible
C306 tolerant
RIL tolerant

C

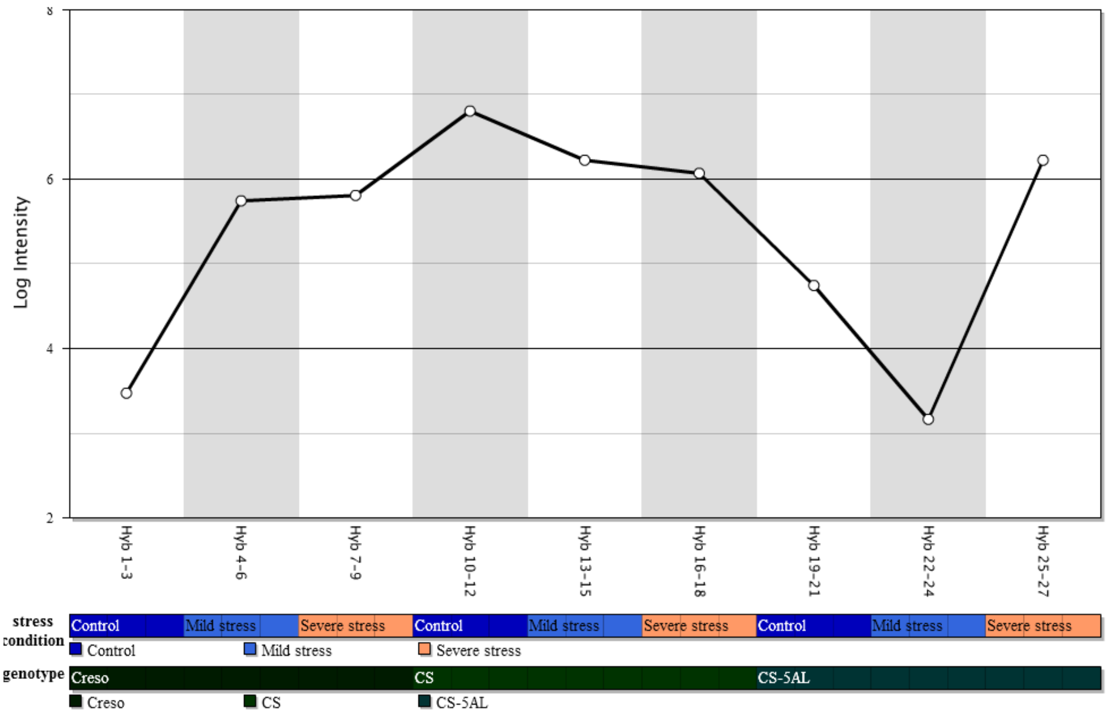

D

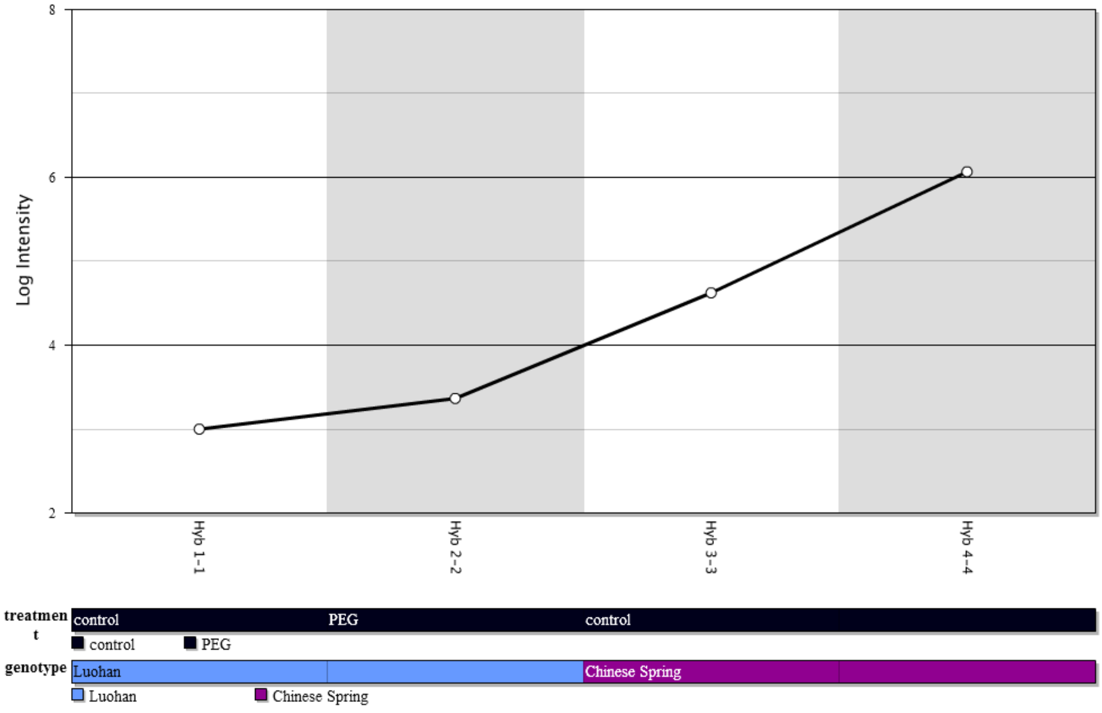

Supplementary Figure S3. The expression profile of Tgt ID Ta.27095.1.S1 (the probe of *TaODORANT1*) in wheat after stresses treatment. A: Drought, heat, and combined stress in durum wheat. B: Transcriptome profiling of reproductive stage flag leaves of wheat in irrigated and drought condition. C: Drought stress in wheat at grain filling stage. D: Drought stress in wheat at grain filling stage (PEG treatment).
